# The emerging potential of autophagy-based therapies in the treatment of cystic fibrosis lung infections

**DOI:** 10.4161/auto.27750

**Published:** 2014-01-13

**Authors:** Robert D Junkins, Craig McCormick, Tong-Jun Lin

**Affiliations:** 1 Department of Microbiology and Immunology; Dalhousie University; Halifax, NS CA; 2 Department of Pediatrics; IWK Health Centre; Halifax, NS CA; 3 Beatrice Hunter Cancer Research Institute; Halifax, NS CA

**Keywords:** autophagy, cystic fibrosis, rapamycin, BECN1, TGM2, *P. aeruginosa*, *B. cepacia*, *H. influenza*, *non-tuberculosis mycobacterium*, *A. fumigatus*, *S. aureus*

## Abstract

Cystic fibrosis (CF) is caused by mutations in the CF transmembrane conductance regulator (CFTR), a channel that normally transports anions across epithelial cell membranes. The most common manifestation of CF is buildup of mucus in the airways and bacterial colonization of the lower respiratory tract, accompanied by chronic inflammation. Antibiotics are used to control CF-associated opportunistic infections, but lengthy antibiotic treatment risks the emergence of multiple-drug resistant (MDR) strains. New antimicrobial strategies are needed to prevent and treat infections in these high-risk individuals. Autophagy contributes to the control of a variety of microbial infections. For this reason, the recent discovery of functional impairment of autophagy in CF provides a new basis for understanding susceptibility to severe infections. Here, we review the role of autophagy in host defense against CF-associated bacterial and fungal pathogens, and survey pharmacologic approaches to restore normal autophagy function in these individuals. Autophagy restoration therapy may improve pathogen clearance and mitigate lung inflammation in CF airways.

## Introduction

Lung damage secondary to chronic infections is the leading cause of morbidity and mortality among patients with cystic fibrosis (CF). Antibiotics are commonly used to prevent and treat CF-associated infections, and have a generally positive track record with respect to improving quality of life and life expectancy. Unfortunately, prolonged antibiotic use can cause undesirable clinical outcomes including the creation of a niche for fungal pathogens which prove extremely difficult to treat and the emergence of multiple drug resistant (MDR) strains of common CF-associated pathogens.^1^^,^^2^ The emergence of MDR strains threatens to undermine the advances made in CF treatment over the past 30 years. For this reason, new therapeutic approaches are needed to prevent and treat CF-associated lung infections.

Etiologic mutations in the CFTR/CF transmembrane conductance regulator contribute to defective immunity and increased lung pathology through a number of mechanisms. Chief among these is osmotic dysregulation in the airway, which results in the accumulation of thick mucus at the surface of respiratory epithelial cells, leading to impairment of pathogen clearance, disruption of TLR/toll-like receptor signaling pathways, and dysregulated inflammatory responses. Protein aggregate accumulation has also been observed in CF airways and a recent landmark study by Luciani, et. al. provided striking molecular evidence that etiologic CFTR mutations trigger a cascade of events that culminate in BECN1/Beclin 1 depletion and impairment of autophagy.^3^ Because autophagy plays an important role in the control of a variety of microbial infections, the functional impairment of autophagy is undoubtedly a significant risk factor for opportunistic infections in CF airways.

Autophagy is an evolutionarily conserved catabolic process through which portions of the cytosol are sequestered and degraded within specialized double-membrane-bound vesicles termed autophagosomes. Over the past decade autophagy has emerged as a central component of the innate and adaptive immune responses where it plays roles in antigen presentation including cross-presentation, direct and indirect killing of intracellular and extracellular pathogens, and the generation of bactericidal peptides.^4^ Autophagy has also emerged as a central regulator of inflammatory responses where it plays roles in modulating inflammasome activation, NFKB activity and interferon production. A growing number of intracellular pathogens have been shown to be specifically targeted to the autophagosome for lysosomal degradation through a process known as xenophagy.^5^ Furthermore, a distinct phenomenon called LC3-associated phagocytosis (LAP) has been implicated in the control of both extracellular and intracellular pathogens. LAP involves many components of the canonical macroautophagy pathway including ATG5, ATG7, LC3, and the BECN1 PtdIns3K complex,^6^ but it does not involve the formation of a double-membrane-bound autophagosome. Instead LAP facilitates processing of phagosomes containing TLR4-, 6-, 9-, or T-cell immunoglobulin mucin 4-associated ligands through the recruitment of LC3 to the phagosomal membrane and delivery of cargo to lysosomes.

There is evidence for autophagy dysregulation in a variety of disease states, including cancer, neurodegenerative diseases, infectious diseases, and autoimmune disorders. For this reason, therapeutic modulation of autophagy is of great interest. Perturbations of autophagy in CF airways suggest that therapeutic strategies aimed at restoring normal autophagy may help prevent and treat CF-associated opportunistic infections. We recently reported a previously unrecognized role for autophagy in host defense against the CF-associated pathogen *P. aeruginosa*, and demonstrated that pharmacological induction of autophagy could enhance clearance of the pathogen in vitro and in vivo.^7^ These findings highlight a growing body of evidence that supports the therapeutic potential of autophagy-inducing compounds, or “autophagy restoration therapy” in combating CF-associated opportunistic infections.

## Cystic Fibrosis as a Disorder Associated with Impaired Autophagy

CF is the most common life-threatening genetic disease in North America and Europe, afflicting approximately 1 in every 3,600 live births.^8^ CF is caused by mutations in the CFTR gene that disrupt anion channel activity. Oxidative stress is a hallmark of CF airways, and recent work has shown that etiologic mutations in CFTR increase the levels of intracellular reactive oxygen species (ROS). One consequence of increased ROS in airway epithelial cells is enhanced activity of TGM2/transglutaminase 2, a calcium-dependent enzyme that creates intra- or intermolecular covalent bonds between proteins by conjugating a reaction between the ε-amino group of a lysine residue and a γ-carboxamide group of a glutamine residue.^9^ In cells bearing etiologic mutations in CFTR, normal ubiquitination and proteasomal degradation of TGM2 is inhibited by ROS-dependent small ubiquitin-like modifier (SUMO)ylation mediated by PIAS4/protein inhibitor of activated STAT, 4.^9^^,^^10^ Inhibited turnover of TGM2, combined with high intracellular concentrations of calcium, drives aberrant TGM2 activity that crosslinks target proteins and results in aggresome formation. Many TGM2 substrates have been identified, but one substrate that directly impacts autophagy is BECN1. TGM2-mediated cross-linking causes sequestration of BECN1 and its accumulation in HDAC6/histone deacetylase 6-, SQSTM1- and ubiquitin-containing cytoplasmic aggresomes. BECN1 sequestration in aggresomes results in the dislodgement of class III PtdIns3K complexes from the endoplasmic reticulum, thereby inhibiting autophagy.^3^

It is not yet known precisely how dysfunctional autophagy leads to the acquisition and persistence of opportunistic lung infections in CF patients, but the functional impairment of autophagy would be expected to undermine innate host defenses by compromising xenophagy and LAP. Therapies targeted at restoring autophagy in cells and animals harboring CFTR mutations restore trafficking of mutant CFTR to the plasma membrane,^3^ attenuate hyperinflammatory responses,^11^ and promote clearance of CF-associated pathogens.^7^^,^^11^^,^^12^ Although the precise molecular details have not yet been fully elucidated, it appears that pharmacologic induction of autophagy may be a useful therapeutic option for CF, and one that may not so easily be thwarted by the emergence of resistance.

## The Role of Autophagy in Host Defense Against Common CF-Associated Pathogens

It has long been recognized that individuals with CF have defects in immunity that render them susceptible to a variety of opportunistic airway infections. Accumulating evidence indicates that autophagy defects could underlie increased susceptibility to infection with certain microbes in CF, particularly those that establish intracellular infections which would normally be cleared by autophagy.^13^ Our current knowledge regarding the role of autophagy in host defense against a variety of CF-associated pathogens is summarized below (Fig. 1).

**Figure:**
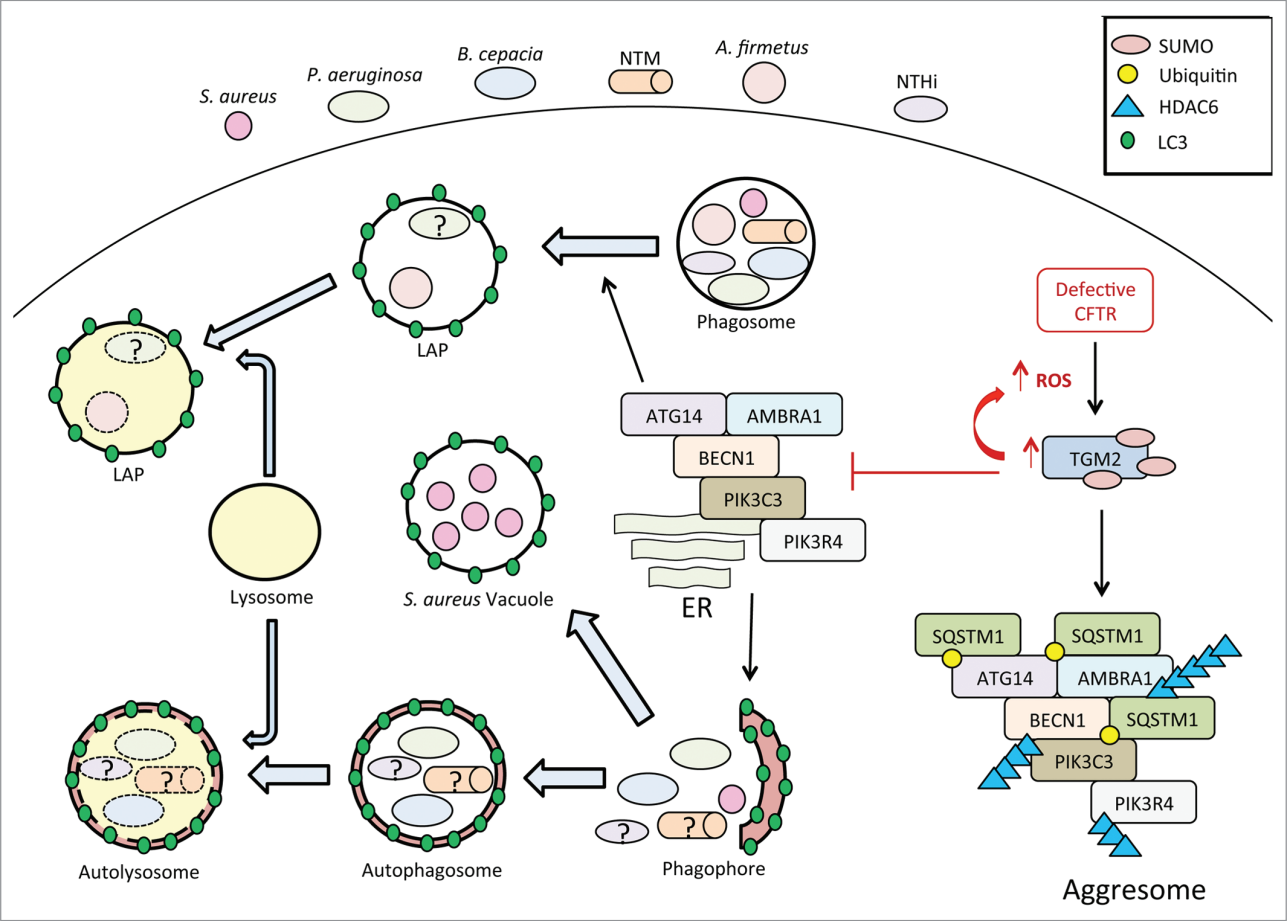
**Figure 1.** Defective CFTR disrupts BECN1 PtdIns3K activity and impairs clearance of CF-associated pathogens. Mutations in the CFTR/cystic fibrosis transmembrane conductance regulator drive increased intracellular levels of reactive oxygen species (ROS) and calcium (Ca^2+^) leading to increased small ubiquitin-like modification (SUMO)ylation of TGM2/ transglutaminase 2. This SUMOylation prevents ubiquitination and proteasomal degradation of the protein, leading to greatly enhanced TGM2 activity, which in turns feeds back to drive ROS production. Increased TGM2 also promotes crosslinking of the BECN1/Beclin-1 phosphatidylinositol 3 kinase (PtdIns3K) complex leading the generation of BECN1, SQSTM1, HDAC6/histone deacetylase 6 and ubiquitin positive aggresomes. Accumulation of BECN1 in aggresomes results in a functional sequestration of the PtdIns3K complex that prevents ER translocation necessary for the initiation of autophagy, or phagosome localization necessary for LC3-associated phagocytosis (LAP). Upon entering the cell, *P. aeruginosa* can become targeted to the autophagy pathway through yet uncharacterized mechanisms. LAP may also play a role in the clearance of intracellular *P. aeruginosa* bacteria. Following phagocytosis *B. cepacia, H. influenzae* and NTM persist within the phagocytic/endocytic pathway where they actively inhibit lysosomal fusion with bacteria containing vesicles. In healthy macrophages, *B. cepacia* containing vacuoles are targeted to the autophagy pathway for degradation. A similar mechanism is involved in the clearance of *M. tubercuolosis*, which employs the same intracellular life cycle as NTMs. However whether or not NTMs are specifically targeted for degradation by the autophagy pathway remains undefined. Similarly, the role of autophagy in the clearance of intracellular *H. influenzae* remains unknown. Following phagocytosis, the degradation of *A. fumigatus* spores requires LAP for effective lysosomal degradation. Unlike the other common CF-associated pathogens, *S. aureus* escapes from the phagosome upon entering the cell. Cytosolic bacteria, or bacteria contained within damaged phagosomes are subsequently targeted to the autophagy pathway where they inhibit lysosomal fusion, creating a replicative niche for the bacteria.

## Pseudomonas aeruginosa

*P. aeruginosa* is the second most common pathogen isolated from CF airways, and MDR strains now infect approximately 10% of all CF patients,^1^^,^^2^ underscoring the need for novel therapeutics. Although largely considered an extracellular pathogen, *P. aeruginosa* can invade host airway epithelial cells where the bacteria can reside for extended periods of time.^14^ It has been proposed that this intracellular phase of infection may be involved in the development of antibiotic resistance and the acquisition of biofilm-like properties which aid the establishment of chronic infection.^14^

In light of these findings, we recently explored the therapeutic potential of pharmacological induction of autophagy in vitro and in vivo in the treatment of acute *P. aeruginosa* lung infection.^7^ We demonstrated in vitro that clearance of intracellular bacteria from human airway epithelial cells was significantly enhanced through induction of autophagy with the mechanistic target of rapamycin (MTOR) inhibitor. Similar observations were made in myeloid-lineage cells that play prominent roles in airway immune responses, alveolar macrophages,^15^ and mast cells,^7^ suggesting that autophagy represents a critical component of the innate immune response against *P. aeruginosa*. Furthermore, we observed that cells harboring the most common mutation in CF patients, ∆F508 CFTR, displayed impaired autophagic responses and failed to clear infection unless pretreated with rapamycin, consistent with the notion that defects in autophagy in CF airways can be pharmacologically reversed. We further demonstrated that pretreatment with rapamycin was able to enhance bacterial clearance in a model of acute *P. aeruginosa* lung infection in vivo. Further work will be required to determine whether pharmacological induction of autophagy will be equally effective in combating established *P. aeruginosa* infections.

The precise role of autophagy in host defense against *P. aeruginosa* remains to be elucidated. *P. aeruginosa* has a type III secretion system that delivers effector proteins into the host cell, including ExoS, an enzyme that inactivates a variety of target host proteins by ADP-ribosylation. ExoS targets include RAB5,^16^ a small GTPase essential for phagolysosome maturation and autophagosome formation.^17^ Thus, ExoS permits invasive *P. aeruginosa* to avoid acidified compartments in epithelial cells, promoting survival.^18^ Our studies demonstrated that *P. aeruginosa* countermeasures could be overcome by rapamycin treatment, but the underlying mechanism of clearance remains obscure. By electron microscopy, we observed bacteria that had clearly been taken up into double-membrane-bound vesicles characteristic of autophagosomes, but these observations were infrequent, suggesting that xenophagy may not significantly contribute to *P. aeruginosa* clearance. It is possible that the enhanced killing of intracellular *P. aeruginosa* following induction of autophagy is actually mediated primarily through LAP, and xenophagy represents a relatively less common event. Our work suggests that *P. aeruginosa* ExoS activity can be at least partially overcome by rapamycin treatment in vivo and in cultured airway epithelial cells and mast cells. Although the mechanistic details regarding the role of autophagy in host defense against *P. aeruginosa* remain to be defined, correcting defects in the autophagy pathway associated with defective CFTR has the potential to restore both xenophagy and LAP, since both processes depend on BECN1-class III PtdIns3K complexes.

## Burkholderia cepacia

*B. cepacia* is an opportunistic bacterial pathogen capable of causing both extracellular and intracellular infections of host epithelial cells and macrophages. Although *B. cepacia* infections are not particularly common in CF patients, afflicting 3–5% of the population,^1^^,^^2^ they are extremely difficult to treat due to multidrug resistance, and because hyperinflammatory responses triggered by the infection accelerate deterioration of pulmonary function, and in some cases lead to fatal necrotizing pneumonia.

The role of autophagy in host defense against *B. cepacia* was recently addressed by Abdulrahman et. al.^12^ It was found that *B. cepacia* becomes targeted to autophagosomes in wild-type macrophages, but not macrophages harboring ∆F508 CFTR mutations, and that the recruitment of the bacteria to these structures targets them for lysosomal degradation. Killing of *B. cepacia* via autophagy could be enhanced through pharmacological induction of the pathway with rapamycin both in vitro and in vivo. Critically, rapamycin was also able to reduce *B. cepacia* induced lung inflammation in a CF mouse model, suggesting that autophagy therapy can both promote clearance of the bacteria from the lungs, and suppress the damaging inflammation responsible for deteriorating lung function and necrotizing pneumonia in CF patients. Considering the unavailability of conventional antibiotic therapies for *B. cepacia* infections, and encouraging pre-clinical results, autophagy-inducing drugs represent a promising therapeutic option.

## Non-Tuberculosis Mycobacterium (NTM)

NTM infections are a growing concern among CF populations due to their increasing prevalence, MDR nature, and because infection is often associated with poor clinical outcomes. Current estimates suggest that NTM strains infect between 5–22% of CF patients.^19^ Among these infections the predominant pathogens were found to be the slow-growing *Mycobacterium avium* complex (MAC) and the fast-growing *Mycobacterium abscessus*. As with *Mycobacterium tuberculosis* (Mtb), the ability to replicate and survive within host cells after infection is a critical determinant of NTM virulence.^20^

Compared with the well-studied role of autophagy in host defense against Mtb, relatively little is known about the role of autophagy during NTM infections. Our only insight comes from a study that noted that prolonged treatment with the antibiotic azithromycin inhibited autophagy and predisposed patients to infection with *M. abscessus*.^21^ In this study impaired autophagy led to decreased clearance of *M. abscessus* from infected macrophages, and the establishment of chronic NTM infection. These findings suggest that as with Mtb, NTM can be targeted for degradation by autophagy. However it is important to note that this study did not address the role of autophagy in host defense against *M. avium* or other NTM strains and further research will be required to fully understand the therapeutic implications of autophagy-inducing drugs in the treatment of these infections.

## Hemophilus influenzae

*H. influenzae* is a small Gram negative bacterial pathogen which includes both encapsulated and unencapsulated strains, the latter of which are referred to as nontypeable *Hemophilus influenzae* (NTHi).^22^ NTHi often establishes biofilms within the lower respiratory tract to chronically colonize the airways of CF patients at a very young age. Although the pathological effects of NTHi infection in CF patients remain incompletely understood, it is thought that the inflammation caused by these infections may predispose the host to later infection with *P. aeruginosa*.^18^

Currently the role of autophagy during host defense against NTHi infection remains undefined. However, a recent report has demonstrated that NTHi can establish long-term intracellular infection of human airway epithelial cells where it remains metabolically active, but nonproliferative.^23^ It has been reported that NTHi bacteria persist in single-membrane-bound vesicles that colocalize with markers of the late endosome, but no colocalization with autophagosomal markers was observed, leading to speculation that autophagy may be actively subverted by as-yet-uncharacterized mechanisms.

## Aspergillus fumigatus

Although the majority of CF-associated infections are caused by bacterial pathogens, the rate of fungal infections with *A. fumigatus* have been steadily increasing and currently affect approximately 20% of all CF patients.^1^ These infections are difficult to treat with conventional antifungal medications, and are associated with profound inflammation in the lungs. In healthy individuals *A. fumigatus* spores are phagocytosed and degraded by alveolar macrophages, neutrophils, and monocytes. *A. fumigatus* has not been shown to invade host cells, but the autophagosomal marker LC3 is recruited to *A. fumigatus*-containing phagosomes, and the recruitment of this protein is essential for effective killing of internalized spores both in vitro and in vivo.^24^ These findings suggest that novel CF therapeutics designed to augment or restore autophagy may effectively target both bacterial and fungal opportunistic infections, achieving “cross-kingdom killing.”

It is important to note that the spores did not appear to become incorporated into classical autophagosomes in this study. Instead, spores were recruited in an ATG5-dependent fashion to LC3-II-decorated single-membrane-bound phagosomes, strongly implicating LAP in spore clearance. The impact of etiologic CFTR mutations on LAP remains incompletely defined, but would be predicted to impair anti-fungal defense. Further study will be required to better understand the role of autophagy defects in the susceptibility to *A. fumigatus* infection in CF airways, and to explore the potential of autophagy promoting therapies in the treatment of fungal infections.

## *Staphylococcus aureus*: A Complication for CF Autophagy Restoration Therapy?

*Staphylococcus aureus* is the single most common CF-associated opportunistic infection, colonizing between 50–68% of the population.^1^^,^^2^ The rate of colonization with methicillin-resistant *Staphylococcus aureus* (MRSA) has skyrocketed over the past decade from just 2% of the CF population in 2001 to more than 25% of the same population in 2010.^2^ MRSA infections in CF patients are associated with decreased lung function, frequent hospitalization, and increased mortality.

*S. aureus* is capable of establishing intracellular infections,^25^ but there is no evidence that it is targeted for autophagic degradation.^26^ Instead, the bacterium escapes the phagocytic pathway and transits to the autophagosome where it prevents lysosomal fusion to establish a replicative niche. Defects in autophagy have previously been reported to prevent *S. aureus* intracellular replication and host cell death.^26^ For these reasons, it is curious that *S. aureus* can so efficiently colonize the CF airway, which clearly has severe impairment of autophagy. A possible explanation for this apparently contradictory data comes from recent reports that demonstrate induction of LC3 conversion and envelopment of bacteria in LC3-positive vesicles is independent of BECN1 and PIK3C3, and instead proceeds through a noncanonical autophagy pathway induced by the *S. aureus* pore forming toxin α-hemolysin and regulated by intracellular levels of cAMP.^17^ These results raise the possibility that defects associated with BECN1 activity in CF may not impact *S. aureus* induced autophagy. Indeed, other studies have demonstrated that *S. aureus* can reside and replicate within the cytosol of airway epithelial cells, and that this process is enhanced by mutations in the CFTR.^27^ Thus, the role of autophagy in CF-associated *S. aureus* infections remains incompletely defined.

Without a more complete understanding of how defects in autophagy induced by mutations in CFTR impact *S. aureus* infection it is difficult to predict how pharmacological manipulation of the pathway might affect the course of infection. Given the ability of intracellular *S. aureus* to commandeer the autophagy pathway to create a replicative niche, it is uncertain whether autophagy-inducing therapy will improve clearance of the bacteria. Furthermore, it will be important to monitor whether the pharmacological induction of autophagy will accelerate *S. aureus*-mediated lung damage. In vitro evidence suggests that induction of autophagy with rapamycin increases numbers of intracellular bacteria, and enhances *S. aureus*-mediated host cell death.^26^ However these effects were only seen at extremely high doses of rapamycin (80 µg/mL) that far exceeded realistic dosage for in vivo studies, and lower doses had no impact on the course of infection.

## Autophagy-Based Therapies

Many of the microbes associated with opportunistic CF airway infections have evolved mechanisms to undermine phagolysosomal maturation and/or autophagy, suggesting that these are potent barriers to be overcome. Reinforcing autophagy via rapamycin treatment has shown some efficacy in promoting clearance of certain microbes in vitro and in vivo, and the exploration of alternative autophagy-inducing drugs is in the early stages. With the recent elucidation of a mechanism underlying defective autophagy in CF airways, and the identification of a drug, cystamine, that can reverse these effects, there is a unique opportunity to regain control over opportunistic infections in CF. What follows is a brief summary of candidate therapeutic approaches intended to restore autophagy in CF airways (Fig. 2).

**Figure:**
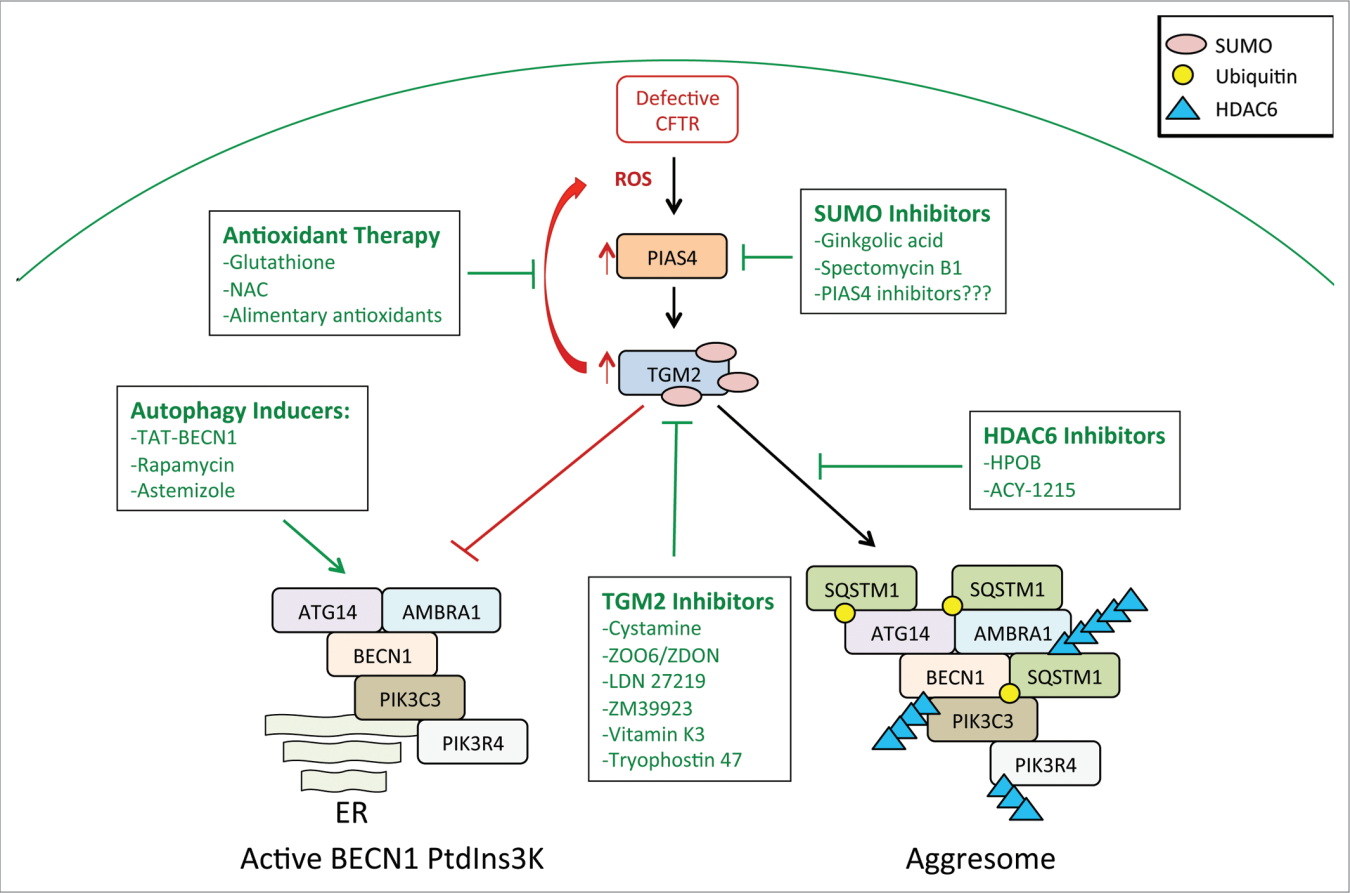
**Figure 2.** Autophagy restoration therapy for CF-associated lung infections. Mutations in CFTR/cystic fibrosis transmembrane conductance regulator result in uncontrolled production of ROS and activation of PIAS4/protein inhibitor of activated STAT, 4 which leads to SUMOylation of TGM2/transglutaminase 2. This post-translational modification results in increased protein stability and activity which drives functional sequestration of BECN1/Beclin 1 in HDAC6/histone deacetylase-6 positive aggresomes resulting in defective autophagy. Aberrant TGM2 activity also perpetuates ROS production resulting in a feedback loop ensuring further activation of TGM2. Therapeutic interventions aimed at restoring autophagy in the airways of CF patients can target multiple facets of this pathway including: 1) Antioxidant therapy to reduce ROS production and disrupt feedback activation of TGM2. 2) Direct autophagy induction to force available BECN1 into active PtdIns3K complexes. 3) TGM2 inhibitors to prevent crosslinking and functional sequestration of BECN1. 4) SUMO or PIAS4 inhibitors to decrease stability and activity of TGM2. 5) Aggresome/HDAC6 inhibitors to prevent the formation of BECN1 containing aggresomes.

## Nonspecific Inducers of Autophagy

One possible approach to treating CF-associated autophagy defects with the aim of improving pathogen clearance and decreasing inflammation in the airways is to employ potent autophagy inducers in an attempt to force newly translated BECN1 into an active PtdIns3K complex before it can be functionally sequestered into HDAC6-positive aggresomes by aberrant TGM2 activity. Therapeutic manipulation of autophagy has become a topic of considerable interest as research has revealed tantalizing links between autophagy and the etiology of many high-priority human diseases. Unfortunately, a limited selection of autophagy-inducing drugs is currently available, many of which suffer from undesirable off-target effects. Current autophagy inducers can be grouped into 3 classes: i) MTOR inhibitors, ii) modulators of calcium dependent signaling or iii) IP3 inhibitors.^28^ Beyond their respective roles in controlling autophagy, each of these targets play integral roles in multiple aspects of cell growth and homeostasis, making them poor targets for the specific induction of autophagy. This issue is highlighted in work exploring the ability of rapamycin, a well-characterized MTOR inhibitor and potent inducer of autophagy, to enhance host defense against CF-associated pathogens. Rapamycin has been shown to promote clearance of the CF-associated pathogens *P. aeruginosa* and *B. cepacia* both in vitro and in vivo.^7^^,^^12^^,^^14^^,^^29^ However in addition to its ability to promote autophagy, rapamycin also has well-characterized immunosuppressive effects. These effects could potentially be beneficial in the treatment of CF-associated lung infection as pathogen-induced lung inflammation is an important cause of decreased lung function in CF patients, and was significantly reduced following rapamycin therapy in vivo.^7^^,^^12^ However the use of immunosuppressive drugs to treat infections could also negatively impact the ability of the patient to fight the infection, and increase their susceptibility to other opportunistic infections. In addition, rapamycin has been associated with significant lung toxicity in transplant recipients.^16^ As a result rapamycin represents a poor candidate for autophagy inducing therapy in the treatment of CF-associated lung infections, and other, more specific autophagy inducers are urgently needed.

To this end, a small molecule screen of 3717 FDA-approved drugs was recently performed in order to identify novel inducers of autophagy.^30^ Among the most effective of these compounds were the anti-psychotic drugs bromperidol, metergoline, thioridazine, and chlorpromazine. The authors demonstrated that these newly identified autophagy inducers were able to significantly reduce IFNG/interferon-γ and LPS induced IL-1β/interleukin-1β production from macrophages, enhanced localization of intracellular *Salmonella* to LC3 positive structures resulting in enhanced bacterial killing, promoted T_reg_ expansion and decreased T_H_17 expansion in vitro. Previous screens have also identified other anti-psychotic drugs which were able to induce autophagy and effectively control mycobacterial infections.^31^ The psychoactive nature of these compounds, and their potentially life threatening side effects, limit their utility, but this work nevertheless provides a strong theoretical basis for future drug development.

A similar drug screen for therapeutic compounds which improve prion disease outcomes unexpectedly identified the second generation selective histamine H_1_-receptor antagonist astmeizole as a potent inducer of autophagy at biologically achievable concentrations.^32^ Astemizole significantly improved survival outcomes in a mouse model of prion disease demonstrating efficacy in vivo. Although the mechanism through which astemizole modulates autophagy, and the ability of astemizole to restore autophagy in CF remain entirely uncharacterized, it represents an attractive option for autophagy induction therapy in CF-associated lung infections due to its previously reported antifungal^33^ and antimalaria^34^ effects. Although not widely used in North America and Europe today due to the availability of superior next-generation histamine receptor antagonists, astemizole has an extremely well-characterized safety and drug interaction profile. In fact, other than the extremely rare occurrence of cardiac arrhythmias associated with astemizole overdose, the drug appears to be relatively safe, and suitable for long-term administration, with appropriate medical supervision.

Fundamental studies of autophagy have also recently led to the development of a novel cell-permeable peptide activator of autophagy.^35^ This peptide was derived from BECN1 and fused to the HIV Tat protein transduction domain; it functions by binding to a newly identified negative regulator of autophagy known as GLIPR2/Gli Pathogenesis-Related 2, and relieving autophagy suppression. Tat-BECN1 peptide has been demonstrated to selectively induce autophagy, improve clearance of protein aggregates, and improve survival outcomes following viral infection in vivo. Although the impact of the peptide has not been tested in a CF model, rescuing BECN1 expression or activity can effectively restore normal autophagy in cells harboring mutation in CFTR, suggesting that this peptide may have some utility in the treatment of CF-associated lung infections.^3^

## Antioxidant Therapy

CF is now well established as a disease characterized by extensive systemic and pulmonary oxidative stress, caused in part by dysregulation of glutathione (GSH) homeostasis and poor adsorption of alimentary antioxidants, which has led to considerable interest in the use of antioxidant therapy in the treatment of the disease (reviewed in ref. 36). Interestingly, in addition to contributing to lung tissue damage, oxidative stress also appears to play a role in defective autophagy in the airways of CF patients. Excessive generation of ROS in the lungs of CF patients has been proposed as a mechanism underlying the crosslinking and functional sequestration of BECN1 by TGM2. Increased TGM2 activity in CF also leads to additional ROS production, creating a feed-forward loop that drives inflammation, tissue damage, and continued dysregulation of the autophagy pathway. For these reasons, antioxidant therapy could significantly reduce inflammation and promote clearance of CF-associated lung infections.

The most well-studied form of CF antioxidant therapy explored to date is direct supplementation with inhaled GSH, or N-acetylcysteine (NAC) which can either be inhaled or delivered orally. The aim is to restore levels of GSH in the lungs to those seen in healthy individuals, where it represents a critical component of antioxidant defenses. Intraperitoneal administration of NAC has been shown to decrease TGM2 SUMOylation, and restore normal levels of autophagy in 2 CF mouse models, supporting the therapeutic potential of antioxidant therapy in the treatment of CF-associated infections.^3^ Small clinical trials have demonstrated that these therapies are safe and generate significant increases in lung GSH levels, and are associated with positive clinical outcomes including decreased lung inflammation, but their efficacy remains to be proven in larger randomized trials. To this end a number of phase II clinical trials are currently underway in the United States and Europe.

Other antioxidant-based therapies have focused on supplementation with various alimentary factors essential for antioxidant defense that are poorly absorbed by CF patients. These factors include the lipid soluble factors vitamin E, carotenoids, coenzyme Q-10, and assorted fatty acids, as well as hydrosoluble factors such as vitamin C, selenium, zinc, and copper. Although preclinical data using these strategies has been promising, to date little is known about their clinical efficacy.

## TGM2 Inhibitors

The most intellectually satisfying way to restore autophagy in CF airways is to design a therapeutic strategy that specifically corrects the defect in autophagy that arises from dysfunctional CFTR. The potential application of the TGM2 inhibitor cystamine in CF patients has recently been reviewed.^37^ Cystamine has been shown to restore normal autophagy in CFTR deficient cells and mouse models.^3^^,^^38^ In addition cystamine has been shown to restore normal trafficking of ΔF508-CFTR and stabilized expression of the protein at the plasma membrane.^38^ The beneficial effects of cystamine treatment persist for extended periods of time after cystamine treatment has ceased, suggesting that once the physiological effects associated with CFTR dysfunction have been corrected, a self-sustaining homeostasis may be re-established.^38^ As a result, cystamine represents a promising avenue for treating opportunistic infections and associated physiological defects in the CF airway.

TGM2 inhibitors are the subject of intense investigation for their potential utility for CF and neurological diseases such as Huntington and Alzheimer diseases.^39^ Importantly, unlike compounds intended for use in neurological disorders, TGM2 inhibitors suitable for use in CF patients will not need to cross the blood-brain barrier. As a result, a re-evaluation of TGM2 inhibitory compounds abandoned previously for their inability to enter the CNS is warranted.

One common approach used to specifically target TGM2 is the chemical modification of natural TGM2 substrates like carbobenzyloxy-L-glutaminylglycine (Cbz-gln-gly). Gluten peptide analogs have also been designed as TGM2 inhibitors, the most promising of which have been dubbed Z006 and ZDON. These inhibitors bind irreversibly in the active site of the enzyme, with promising results in vitro, although their efficacy in vivo has not yet been thoroughly examined. Finally, recent screens have identified a host of compounds including LDN 27219, the Janus kinase 3 inhibitor ZM39923, its metabolite ZM449829, tyrphostin 47, and vitamin K3, as potent inhibitors of TGM2. Again the in vivo efficacy of these compounds remains to be proven. However the continued efforts to design potent and specific TGM2 inhibitors could provide exciting opportunities over the coming years to disrupt aberrant BECN1 aggresome formation, restoring normal autophagy, and potentially significantly ameliorating persistent infections and inflammation in CF airways.

## SUMOylation Inhibitors

TGM2 SUMOylation is essential for enhanced enzyme activity and protein levels in the airways of CF patients.^10^ Elevated levels of ROS leads to greatly increased expression of PIAS4, the SUMO E3 ligase responsible for TGM2 SUMOylation. Disruption of the SUMOylation machinery has been shown to be sufficient to restore TGM2 homeostasis in CF cell lines providing a theoretical basis for the use of SUMOylation inhibitors in the treatment of CF-associated lung infections. A number of SUMOylation inhibitors have been identified to date, including ginkgolic acid and spectomycin B1, which target SUMO E1 and E2 intermediates respectively. Although the theoretical basis for anti-SUMOylation therapy has been established in many human diseases, the ultimate applicability of such therapies remains to be proven. SUMOylation is a critical regulator of multiple aspects of cell biology including nuclear-cytosolic protein transport, transcription regulation, protein stability and degradation, stress responses, and cell cycle progression. Furthermore, SUMOylation has been implicated both in the normal progression and the dysregulation of CFTR biogenesis and quality control.^40^ Due to these essential roles of SUMOylation in various cellular processes including CFTR processing, direct inhibition of the pathway may be associated with considerable off target effects. An alternative drugable target may instead be PIAS4, the enzyme responsible for aberrant SUMOylation of TGM2 in CF airways. Although no specific PIAS4 inhibitors have been identified to date, gene silencing of the enzyme is sufficient to restore TGM2 homeostasis in vitro. As a result, this therapeutic approach to the treatment of CF-associated lung infections, as well as the development of specific PIAS4 inhibitors, warrants further consideration.

## Aggresome Inhibitors

A key event in the disruption of the autophagy pathway in CF airways is the formation of insoluble, HDAC6 positive aggresomes that functionally sequester the BECN1 PtdIns3K complex. Thus, preventing aggresome formation may help to maintain the active pool of BECN1. Evidence for the use of HDAC6 inhibitors to prevent aggresome formation can be found from studies demonstrating that siRNA silencing of HDAC6 abolishes aggresome formation in pancreatic cancer cells.^41^ Although first generation HDAC6 inhibitors such as tubacin and tubastatin were unsuitable for clinical trials, the recent description of second generation inhibitors such as N-hydroxy-4-(2-[(2-hydroxyehtyl)(phenyl)amino]-2-oxoethyl)benzamide (HPOB)^42^ and ACY-1215^43^ paves the way for selective inhibition of aggresome formation in vivo.

Although HDAC inhibitors may aid in restoring BECN1 homeostasis in the airways of CF patients, other factors need to be taken into account when considering the therapeutic potential of such interventions. First, although specific HDAC6 inhibitors have not yet been tested clinically, other HDAC inhibitors are associated with considerable hematological and gastrointestinal toxicity in cancer patients. Furthermore, HDAC inhibitors have been shown to suppress the innate immune response, both by inhibiting inflammatory responses and by increasing susceptibility to bacterial and fungal infections.^44^ Finally, HDAC6 has been implicated in autophagosome-lysosome fusion, and HDAC6 deficiency leads to autophagosome maturation failure and aggresome accumulation.^45^ Hence inhibition of HDAC6 may lead to secondary disruption of the autophagy pathway, and further increase susceptibility to infection in the airways of CF patients. As a result, future investigation will be essential in order to determine the efficacy and safety of HDAC6 inhibitors in the treatment of autophagy defects associated with CF.

## Conclusions

The characterization of etiologic defects in autophagy associated with CFTR mutations has greatly advanced our understanding of immune dysfunction in CF. Although much work remains to be done to fully elucidate the role of autophagy in the control of myriad CF-associated pathogens, early studies suggest that “autophagy restoration therapy” is worthy of thorough investigation. Proof-of-principle studies have demonstrated rapamycin-induced clearance of CF-associated pathogens and concomitant decreased pathogen-induced lung inflammation. However, a complex interplay exists between both pathogenic and nonpathogenic commensal microorganisms in CF airways. For this reason, a systematic survey of the microbiome of normal and CF airways is warranted, and candidate autophagy-inducing drugs will have to be carefully evaluated for their impact on pathogens and benign commensal organisms.

Although the paucity of specific autophagy-inducing drugs limits the clinical potential of this treatment strategy at this time, the nature of the defects leading to impaired autophagy in CF provides a variety of potential targets for therapeutic intervention. Many of the autophagy-restoring therapeutic strategies outlined above could be employed in combination with conventional antibiotic therapies to target intracellular and extracellular pathogens simultaneously, accelerating eradication of opportunistic infections in CF airways.

## Abbreviations:

CF: cystic fibrosis
CFTR: cystic fibrosis transmembrane conductance regulator (ATP-binding cassette sub-family C, member 7)
GSH: glutathione
HDAC: histone deacetylase
LAP: LC3-associated phagocytosis
MDR: multiple-drug resistant
MRSA: methicillin-resistant *Staphylococcus aureus*
Mtb: *Mycobacterium tuberculosis* (Mtb)
MTOR: mechanistic target of rapamycin
NAC: N-acetylcysteine
NTHi: nontypeable *Haemophilus influenza*
NTM: nontuberculosis mycobacterium
PtdIns3K: phosphatidylinositol 3 kinase
PIAS4: protein inhibitor of activated STAT, 4
SUMO: small ubiquitin-like modifier
TGM2: transglutaminase-2
TLR: toll-like receptor

## Notes

10.4161/auto.27750

## Notes

JunkinsRDShenARosenKMcCormickCLinTJ Autophagy enhances bacterial clearance during *P. aeruginosa* lung infection PLoS One 2013 8 e72263 10.1371/journal.pone.0072263
